# Temporal-responsive hydrogels reprogramming energy metabolic pathway in the bone-angiogenic cascade for diabetic bone regeneration

**DOI:** 10.1016/j.mtbio.2026.103394

**Published:** 2026-06-24

**Authors:** Kai Jiang, Kai Wang

**Affiliations:** a Department of Critical Care Medicine, Sichuan Provincial People's Hospital, University of Electronic Science and Technology of China, Chengdu, 610054, China; b Department of Spine Surgery, Honghui Hospital, Xi'an Jiaotong University, Xi'an, 710054, China

**Keywords:** Temporal hydrogels, Mitochondria, Vascularization, Diabetes, Bone regeneration

## Abstract

Diabetes-associated metabolic dysregulation disrupts the angiogenic–osteogenic cascade required for bone healing by impairing oxygen delivery and suppressing mitochondrial oxidative phosphorylation. Here, transcriptome sequencing data reveal significant temporal differences in energy metabolism between early and late diabetic bone defects. Guided by this temporal energy demand, we developed an injectable temporal-responsive hydrogel (TRH) integrating continuously released strontium-doped hydroxyapatite (Sr-HA) with berberine (Ber)-loaded chitosan microspheres for delayed mitochondrial regulation. The temporal release profile closely matches the metabolic transition during bone regeneration. Continuous Sr^2+^ release rescued endothelial migration and network formation under hyperglycemia, whereas sustained Ber release was associated with activation of the AMPK/PGC-1α-related mitochondrial pathway, restoration of mitochondrial membrane potential and ultrastructure, and improved ATP production to support osteogenic differentiation and mineralization. In a critical-sized calvarial defect in diabetic mice, TRH markedly increased bone volume and improved trabecular microarchitecture, accompanied by enhanced CD31-positive vascularization, OCN/OPN-mediated osteogenesis, and elevated PGC-1α signaling. This work therefore provides a temporally programmed metabolic intervention that restores angiogenesis and osteogenesis for diabetic bone regeneration.

## Graphical abstract

**Figure:**
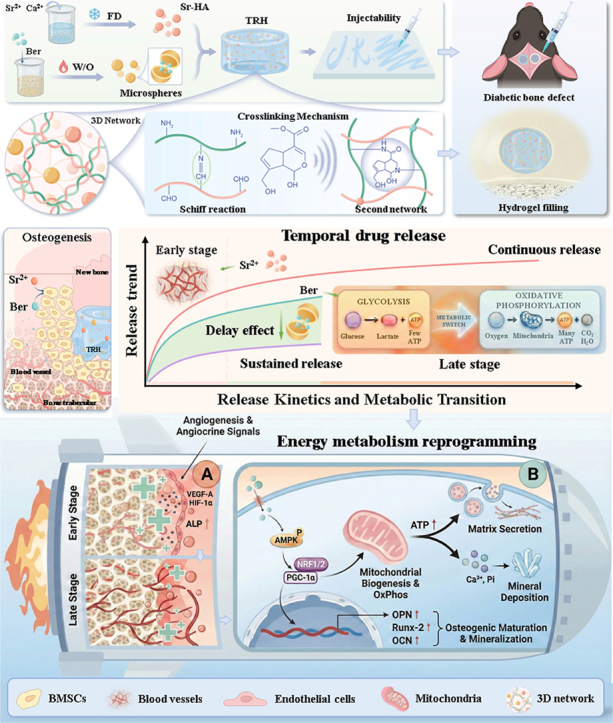

## 1 Introduction

Dysregulation of energy metabolic pathways is one of the key factors limiting vascular–bone cascade regeneration in patients with diabetes [[1], [2], [3]]. Bone repair is a complex and temporally orchestrated biological process that involves hematoma inflammation, soft callus formation, hard callus formation, and bone remodeling [[4], [5], [6]]. These essential biological activities are powered by cellular energy metabolism [7,8]. Recent reviews have further emphasized that bone metabolism and homeostasis are also regulated by post-transcriptional mechanisms such as alternative splicing and extracellular vesicle-mediated intercellular communication [9,10]. Compared with normal bone defects, patients with diabetes experience delayed bone regeneration following injury due to intrinsic dysregulation of glucose metabolism, which leads to mitochondrial dysfunction, enhanced glycolysis, and impaired oxidative phosphorylation [11,12]. In addition to glucose metabolism, amino-acid metabolic programs, including tryptophan metabolism, have also been implicated in osteochondral homeostasis [13]. The biodegradable iron-capturing hydrogel promotes cranial defect regeneration by releasing mitochondrial-targeted iron chelator nanoparticle, which specifically targets and chelates mitochondrial iron ions, thereby correcting abnormal energy metabolism [14]. Similarly, a melatonin-loaded gelatin/Polylactic acid nanofiber scaffold fabricated through a stepwise manufacturing strategy stimulates mitochondrial activity in bone marrow mesenchymal stem cells (BMSCs), as reflected by upregulated respiratory chain factors. The resultant improvement in energy metabolism promotes vascularization, thereby opening the material transport pathways essential for bone regeneration [15]. Current research indicates that bioactive materials capable of modulating energy metabolism exert a positive effect on the bone repair process [16,17]. However, a comprehensive investigation of the temporal transitions in energy metabolism during the physiological process of bone regeneration, and the development of material-based strategies to address the energy demand challenges in the vascular–bone cascade regeneration, remain significant challenges.

The essence of the temporal dynamics in energy metabolism is closely tied to the state of cellular differentiation. In previous studies investigating the role of energy metabolism in bone repair, the energy metabolism during the bone repair process has typically been treated as a continuous and invariable whole for discussion [18,19]. However, such simplified treatment may obscure the dynamic patterns of energy demand across different stages, thereby imposing certain limitations on a deeper understanding of the energy metabolism mechanisms involved [19,20]. Indeed, during the recruitment phase of mesenchymal stem cells, energy is primarily supplied rapidly through glycolysis, a stage characterized by active cell proliferation but low oxygen consumption [21,22]. As the process transitions to the differentiation phase, mitochondrial oxidative phosphorylation becomes more critical, particularly requiring substantial ATP for the expression of alkaline phosphatase [6,23]. By the mineralization phase, the transport of calcium and phosphate ions and crystal deposition further elevate energy demands. In the early stages of repair, promoting angiogenesis to improve local oxygen supply can lay the groundwork for subsequent phases with high energy requirements [[24], [25], [26]]. In recent years, the development of temporally controlled delivery systems has provided new approaches to address this issue. The Janus fibrous membrane fabricated via electrospinning and layer-by-layer self-assembly enables the sequential release of growth factors, achieving rapid release of aFGF and sustained release of BMP-2. Enhanced oxidative phosphorylation accelerates the osteogenic differentiation of BMSCs, thereby aligning with the temporally energy demands of different repair stages [7]. Notably, enhanced oxidative phosphorylation not only directly supplies energy but also accelerates the osteogenic differentiation of BMSCs. Based on the above analysis, we propose the following scientific hypothesis: temporal regulation of energy metabolism represents a core strategy to overcome impaired bone repair in diabetes. By targeting the metabolic reprogramming during the vascular-osteogenic repair stages, the efficiency of bone regeneration under diabetic conditions can be enhanced.

The design and development of temporal-responsive materials open a highly promising avenue for the precise regulation of bone tissue regeneration [[27], [28], [29]]^.^ Smart hydrogels have also been summarized as microenvironment-modulating platforms for spatially and temporally controlled bone reconstruction [30]. Enzyme-responsive DNA-PEG smart hydrogel provides a programmable therapeutic cascade approach. In the early stage, matrix metalloproteinase‐triggered hydrogel degradation releases VEGF, initiating angiogenesis and promoting osteogenesis. In the later stage, nuclease‐mediated DNA cleavage liberates phosphate groups. This process works in concert with endogenous calcium ions to drive mineralization, thereby achieving spatiotemporal regulation of bone repair [31]. Similarly, a sequential delivery system composed of GelMA hydrogel combined with PLA microspheres enables rapid release of nerve growth factor (NGF) and delayed release of Yoda1, emulating the temporal pattern of natural bone repair and providing a precise therapeutic strategy for bone defects [32]. Current research predominantly focuses on the temporal variations of material metabolism in physiological processes, while the temporal role of energy in bone repair has been largely overlooked. The precise control of energy can more effectively drive distinct physiological processes during bone repair [33,34]. Since matter serves as the carrier of energy, it is theoretically possible to achieve temporal control over energy release by modulating the metabolism and transformation of matter [35,36]. In addition, in situ fabrication technology is a method for forming materials within a living environment, offering advantages such as minimal invasiveness, biocompatibility, biodegradability, and personalized customization [37,38]. Integrating the design concept of temporal materials with in situ fabrication technology provides a transformative paradigm for developing bone tissue engineering materials capable of temporal energy regulation.

**Fig. 1:**
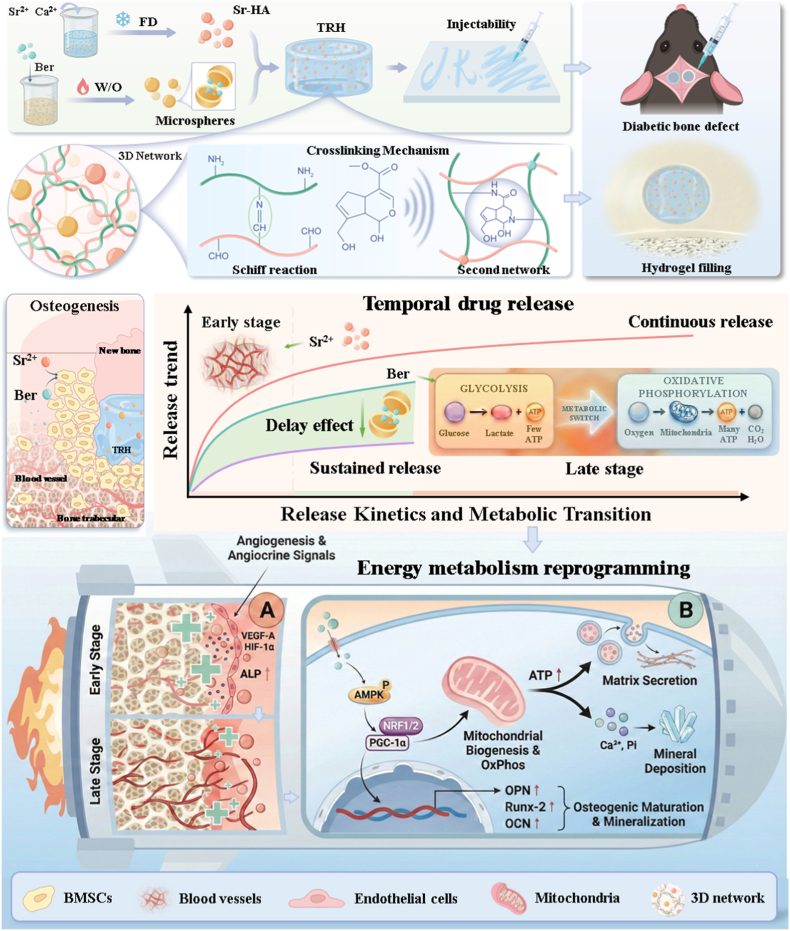
Fabrication schematic of temporal-responsive hydrogels (TRH) and their application in the bone-angiogenic cascade for diabetic bone regeneration by reprogramming energy-metabolic pathways. TRH contains Sr-HA, which promotes angiogenesis, and Ber-loaded microspheres that support mitochondrial regulation through the AMPK/PGC-1α-related pathway. Temporal drug release is designed to match the metabolic requirements of bone repair and to promote diabetic bone regeneration.

Based on the above analysis, we performed transcriptome sequencing to uncover the dynamic evolution of energy metabolism during diabetic bone defect repair and subsequently proposed a temporal energy-sustaining strategy. We therefore designed and prepared an injectable temporal-responsive hydrogel (TRH) that simultaneously loads strontium-doped hydroxyapatite (Sr-HA) and berberine (Ber)-encapsulated chitosan microspheres. TRH is designed to promote vascular regeneration through Sr^2+^ release, while sustained Ber release provides mitochondrial metabolic support during later osteogenic maturation. This coordinated action harmonizes the vascular-osteogenic cascade, thereby accelerating diabetic bone defect repair (Fig. 1). In vitro experiments demonstrate that early Sr^2+^ release initiates vascularization and supports early osteogenic priming. Subsequently, sustained Ber release may contribute to late-stage osteogenic maturation by supporting the PGC-1α-related mitochondrial program, improving oxidative metabolism and ATP availability. Sequencing results suggest that TRH reshapes energy metabolism-related pathways and provides energetic support for bone regeneration. In the critical-sized diabetic bone defect model, TRH showed marked therapeutic potential, offering a promising strategy for diabetic bone regeneration.

## 2 Results and discussion

### 2.1 Temporal analysis of energy metabolism in diabetic bone defect

**Fig. 2:**
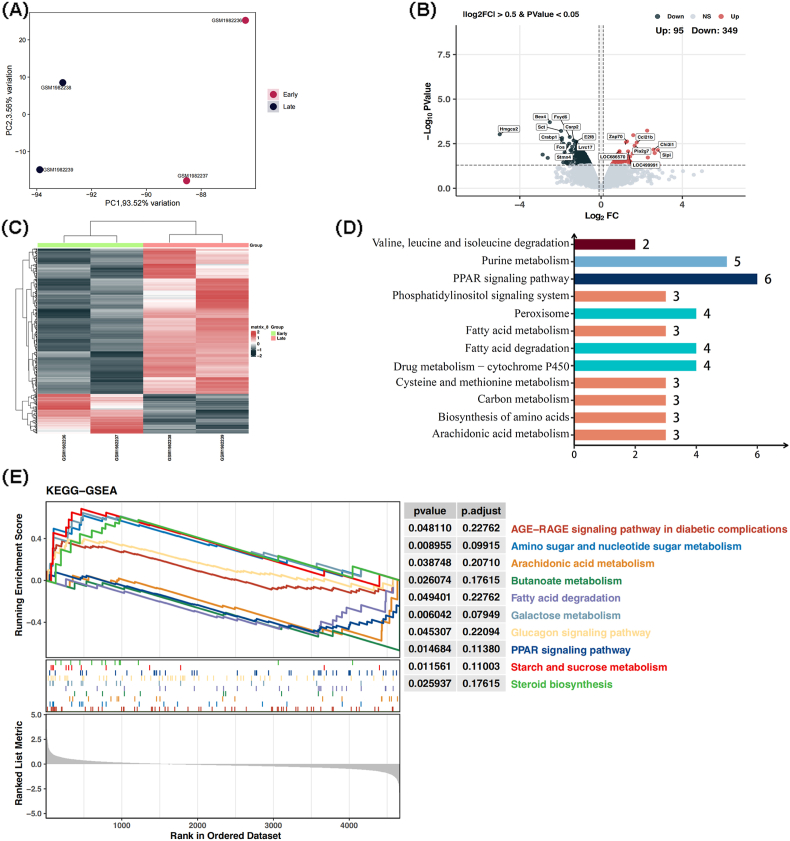
Transcriptomic analysis of diabetic bone defect tissues at different repair stages in GSE76364. Day 5 fracture callus samples were defined as the early repair stage, and day 11 samples were defined as the later repair stage. (A) PCA clustering based on transcriptome data. (B) Volcano plot showing differential gene expression between the later- and early-stage groups. (C) Heatmap of differential gene expression; magenta and green color intensities represent gene upregulation and downregulation, respectively. (D) KEGG pathway comparative analysis and enrichment analysis of differential genes. (E) GSEA pathway enrichment analysis results of differential genes.

An in-depth analysis of the transcriptomic sequencing data from diabetic bone defect samples revealed significant transcriptome remodeling between different repair stages. Specifically, the public dataset GSE76364 contains fracture callus samples collected at post-fracture day 5 and day 11; these were defined in this study as the early and later repair stages, respectively. Principal component analysis showed a clear separation between the two stages along PC1 (variance contribution 93.52%), indicating profound alterations in the overall gene expression profile during diabetic bone repair (Fig. 2A). Differential expression analysis identified 444 significantly differentially expressed genes, of which 95 were upregulated and 349 were downregulated in the later-stage group (Fig. 2B). Heatmap analysis further demonstrated that these genes exhibited highly consistent expression patterns between the two groups, indicating a close association with the repair stage (Fig. 2C). KEGG pathway enrichment analysis identified multiple metabolic and signaling pathways that were significantly altered (P < 0.05). Among these, enrichment of the PPAR signaling pathway suggests that regulation of lipid metabolism and insulin sensitivity may be impaired in the later stage, which is often associated with energy metabolism reprogramming and dysregulated inflammatory control.

The bar chart illustrates the distribution of upregulated and downregulated gene counts across various pathways (Fig. 2D). GSEA results extend the above findings. The enrichment score plot and the ranked list metric plot clearly identify specific pathways that are significantly activated in the late stage, with the AGE-RAGE signaling pathway being particularly prominent (Fig. 2E). This pathway is typically driven by the accumulation of glycation end products under hyperglycemic conditions. It can activate downstream inflammatory signals such as NF-κB, exacerbating oxidative stress and cellular dysfunction. This mechanism is likely directly linked to the sustained inflammatory response and impaired repair in diabetic bone defects. Other significantly enriched pathways also include signaling modules related to extracellular matrix metabolism and immune-inflammatory responses. The table on the right lists the statistical parameters (p-value and FDR) corresponding to these pathways, thereby systematically revealing the core functional characteristics and potential regulatory networks that distinguish early from late stages of diabetic bone defects.

Transcriptomic analysis indicated significant molecular reprogramming between the early and later stages of diabetic bone defect healing. The large number of differentially expressed genes revealed by PCA suggests that energy metabolism in diabetic bone defects changes dynamically during repair. Enrichment of metabolic pathways among the differentially expressed genes, particularly the PPAR signaling pathway, a critical regulator of lipid metabolism and energy homeostasis, indicates a fundamental shift in energy-substrate utilization. The significant enrichment of the AGE-RAGE signaling pathway identified by GSEA is also notable. Its prominence at the later stage suggests a progressively detrimental microenvironment that may disrupt the energy-production mechanisms required for bone repair. Overall, these gene-expression patterns suggest that later-stage defect repair may be characterized by insufficient metabolic adaptation or a shift toward a low-efficiency metabolic state, with persistent glycolytic activity. Such metabolic dysregulation may contribute to impaired cell proliferation, differentiation, and matrix synthesis in diabetic bone disease, thereby leading to delayed healing or non-union.

### 2.2 Preparation and characterization of temporal hydrogels

**Fig. 3:**
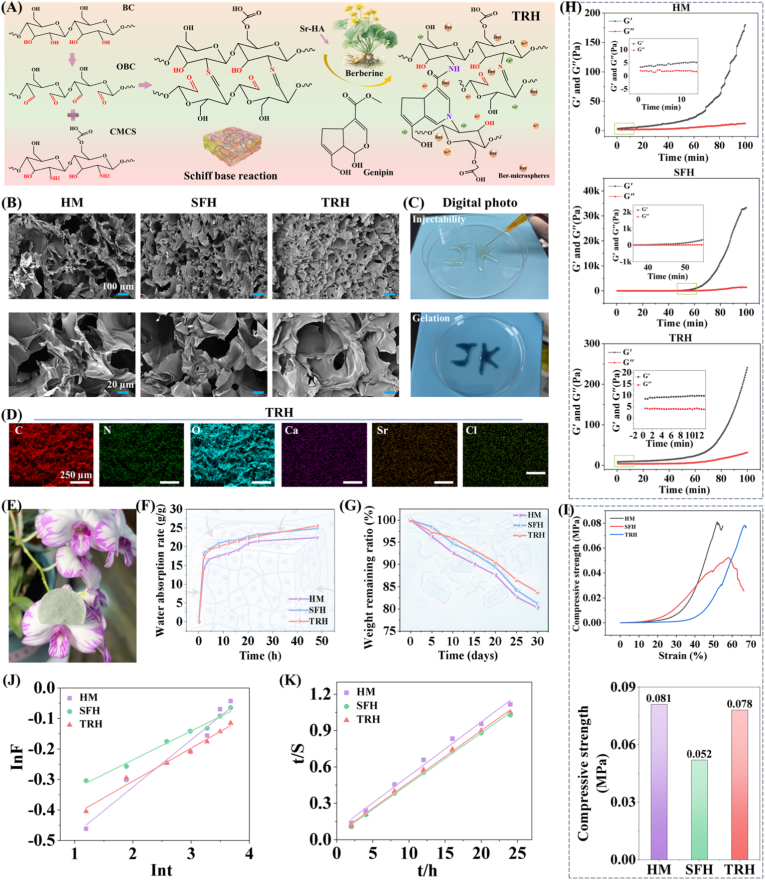
Preparation and characterization of temporal hydrogels. (A) The formation mechanism of hydrogels. (B) SEM images and (C) digital photo about injectability of hydrogels. (D) EDS images of TRH. (E) TRH on the stamens. (F) Water absorption rate in the first 48 h. (G) Degradation behavior of hydrogels in vitro. (H) Evolution of storage modulus (G′) and loss modulus (G″) with time. (I) Compression test results of temporal hydrogels. (J) Relationship diagram of ln F ∼ ln t and (K) t/S ∼ t of the swelling process of temporal hydrogels.

Fig. 3A illustrates the molecular changes during the formation of temporal-responsive hydrogels. The hydrogel matrix is formed by oxidized bacterial cellulose and carboxymethyl chitosan through a Schiff base reaction, producing the first dynamic network. Subsequently, a robust second network is established via crosslinking by the crosslinker genipin. Berberine-loaded chitosan microspheres and Sr-HA particles are well encapsulated within the matrix, thereby constructing the temporal hydrogel TRH. The microstructure of the hydrogels reveals that all three types possess abundant porous architectures with interconnected pores, which provide structural support for the release of functional particles (Fig. 3B). It is noteworthy that the pore structure of TRH is denser than that of HM and SFH. Such a compact structure can better retard the release of functional particles, thereby contributing to the temporal drug release performance of the material.

Under ambient temperature conditions, TRH can be smoothly injected through a syringe with continuous flow and no needle blockage. After injection, the precursor solution completes in situ gelation within 6 h, forming a uniform three-dimensional network structure (Fig. 3C). EDS analysis reveals the encapsulation of functional particles within TRH, with Ca, Sr, and Cl elements showing a highly uniform distribution (Fig. 3D). After freeze-drying, TRH can be supported by a flower stamen, indicating its lightweight nature and high drug-loading efficiency, which are highly beneficial for bone repair (Fig. 3E). Next, we analyze the water absorption and degradation properties of the hydrogels. At 48 h, the water absorption ratios of HM, SFH, and TRH are 22.4 g/g, 24.8 g/g, and 25.6 g/g, respectively (Fig. 3F). At day 30, the weight remaining ratios of HM, SFH, and TRH are 80.2%, 81.2%, and 83.8%, respectively (Fig. 3G). It is observed that the three samples show little difference in both water absorption and degradation rates, which is closely related to the consistency of their gel matrices.

The rheological results show that G′ was consistently higher than G″ for all hydrogels, indicating the formation of elastic-dominant gel networks (Fig. 3H). The formation of the sequential hydrogel involves the establishment of two network structures. When oxidized bacterial cellulose was mixed with carboxymethyl chitosan, a dynamic Schiff base network was formed; therefore, the phenomenon of G′ > G″ was observed for HM. After the addition of Sr-HA, the gelation time of SFH was prolonged in the initial stage, which may be attributed to the fact that Sr-HA did not participate in the crosslinking, thereby disrupting the dynamic Schiff base network. However, in TRH, the incorporation of berberine-loaded chitosan microspheres provided additional binding sites, thereby restoring its initial gelation time. For HM, SFH, and TRH, G′ ≫ G″ at the later stage, which is attributed to the formation of a robust second network established by the crosslinker genipin. In the compression test, the maximum compressive strengths of HM, SFH, and TRH were found to be 0.081, 0.052, and 0.078 MPa, respectively, with corresponding strains of 51.9%, 58.2%, and 66.7% (Fig. 3I). The compression results further corroborated the rheological data, reflecting intrinsic mechanisms consistent with the observed rheological properties.

Furthermore, the Korsmeyer–Peppas kinetic model and the quasi-second-order kinetic model were employed to investigate the swelling kinetics of the hydrogels. In the Korsmeyer–Peppas model, the R^2^ values for HM, SFH, and TRH were 0.9591, 0.9860, and 0.9849, respectively (Fig. 3J). As shown in the table (Table S2.), the n values of all hydrogels were below 0.5, indicating that the swelling process of the hydrogels followed typical Fickian diffusion in theory. In the quasi-second-order model, the R^2^ values for HM, SFH and TRH were 0.9921, 0.9983, and 0.9982, respectively (Fig. 3K and Table S3.). The swelling behavior of the hydrogel was better fitted by the quasi-second-order model, indicating that the swelling process was not governed solely by Fickian diffusion, but was also closely related to polymer chain relaxation, network rearrangement, and interactions between water molecules and hydrophilic groups within the hydrogel matrix. In short, we successfully construct an injectable temporal hydrogel incorporating berberine-loaded chitosan microspheres and Sr-HA particles.

### 2.3 Investigation on the temporal material basis and properties of hydrogels

**Fig. 4:**
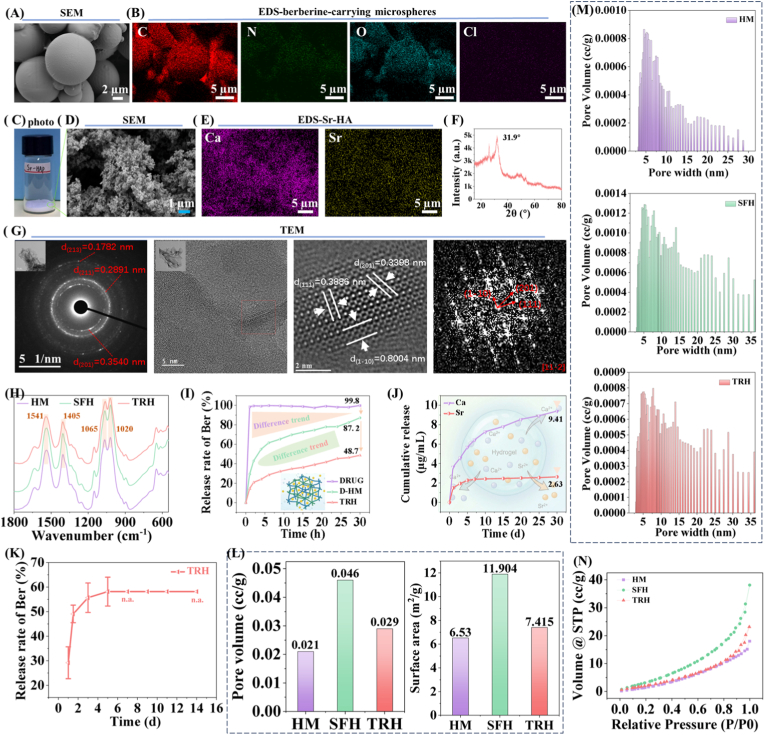
Investigation into the temporal material basis and properties of hydrogels. (A) SEM images and (B) EDS images of berberine-carrying microspheres. (C) The Digital photo, (D) SEM, (E) EDS, (F) XRD, and (G) TEM of nano-particle Sr-HA. (H) FTIR spectra of all scaffolds. (I) Cumulative release rate of Ber within 30 h using UV–vis. (J) Cumulative release rate of Sr and Ca. (K) Cumulative release rate of Ber within 14 d using HPLC, n.a. = not detected. (L) Pore volume and surface area of hydrogels. (M) Pore size distribution of hydrogels. (N) N_2_ sorption/desorption isotherms.

The foundation of energy temporality lies in the temporality of matter. The intrinsic connection between the internal structure of materials and their macroscopic properties determines the temporal variation patterns of hydrogels. The basic components of temporal-responsive hydrogels components warrant further analysis to elucidate the temporal release of drugs. Fig. 4A shows that the berberine-loaded microspheres exhibit a regular spherical shape, with a smooth and dense surface structure, and no obvious pores or topological roughness. EDS analysis further demonstrates that Cl is present, indicating the successful encapsulation of Ber within the TRH (Fig. 4B). As the second component of the temporal-responsive hydrogels, Sr-HA appears as a white solid powder (Fig. 4C), composed of aggregated nanoscale particles (Fig. 4D). Representative elements Ca and Sr are observed in the EDS analysis, preliminarily confirming that the synthesized mineral is strontium-doped hydroxyapatite (Fig. 4E). A sharp characteristic peak is observed at 31.9° in Sr-HA (Fig. 4F), according to JCPDS card number 09-0432 [35], which is a characteristic peak of hydroxyapatite, indicating that the mineral component possesses the characteristic structure of hydroxyapatite. In TEM diffraction mode, diffraction rings of the crystal structure are observed. The characteristic crystal plane (211) of hydroxyapatite is identified, with an interplanar spacing of 0.2891 nm (Fig. 4G). High-resolution crystal phases are further analyzed. Lattice fringes with interplanar spacings of 0.3886 nm, 0.8004 nm, and 0.3398 nm are observed, corresponding to the (111), (1-10), and (201) crystal planes, respectively. FTIR results show that the curves of HM, SFH, and TRH are consistent (Fig. 4H). This is because the gel matrices of the three samples are made of the same material and underwent the same processing. Characteristic peaks of the carboxymethyl chitosan backbone are observed at 1065 cm^−1^ (C-C) and 1020 cm^−1^ (C-O). Simultaneously, a characteristic peak corresponding to the C=N bond is observed at 1541 cm^−1^, confirming the successful formation of the genipin-crosslinked gel.

To investigate the temporal-responsive release characteristics of the drug, we designed the following comparative experiments: the release behavior of berberine directly added to PBS (DRUG), the drug release behavior of a berberine-loaded hydrogel matrix in PBS (D-HM), and the drug release behavior of TRH in PBS. The difference trend between D-HM and DRUG gradually decreased over time, and at 30 h, the cumulative release rate reaches 87.2%. TRH exhibits a slow-release rate throughout the entire period, showing a cumulative release of only 48.7% at 30 h (Fig. 4I). We hypothesize that the time-dependent nature of TRH release is due to the different pathways the drug takes during its release. The drug is not only hindered by the gel matrix, but its release from the microspheres further delays the release time. This temporal difference in release patterns aligns perfectly with our design intentions. Osteogenic differentiation is a relatively long-term process, particularly during the late stage (day 14). Therefore, HPLC was employed to determine the release of berberine (Fig. 4K). The cumulative release of berberine reached 49.1% on day 1.5 and continued to increase until day 5, reaching 58.2%. However, no further release of berberine was detected in the subsequent experiments. The release of berberine was evaluated using two methods (UV–vis and HPLC), which yielded slight numerical differences but showed a consistent overall trend. Subsequently, we tested the release of functional Ca^2+^ and Sr^2+^. Both Ca^2+^ and Sr^2+^ can be released continuously for up to 30 d. On day 30, the release of Ca^2+^ and Sr^2+^ is 9.41 and 2.63 μg/mL, respectively (Fig. 4J). The short-term release of berberine (5 d) and the long-term release of functional ions (30 d) were well aligned with the osteogenic differentiation cycle.

The porous structure of the hydrogel plays a regulatory role in drug release. The pore volumes of HM, SFH, and TRH were 0.021, 0.046, and 0.029 cc/g, respectively. After the incorporation of Sr-HA, the structure of SFH was disrupted, leading to decreased mechanical properties and an increased pore volume. After the drug-loaded microspheres were incorporated, the number of crosslinking sites increased, the degree of crosslinking in TRH was enhanced, the mechanical properties were restored, and the pore volume consequently decreased. The surface areas of HM, SFH, and TRH showed a similar trend, with values of 6.53, 11.904, and 7.415 m^2^/g, respectively (Fig. 4L). In the pore size distribution, the pore width of HM was mainly below 10 nm. After the functional particles Sr-HA and the berberine-loaded chitosan microspheres were incorporated, the number of pores in the 15–35 nm range increased significantly (Fig. 4M). This is also an intrinsic factor contributing to the higher surface areas of SFH and TRH compared with HM. The adsorption and desorption curves nearly overlapped, indicating that HM、SFH and TRH exhibited good reversibility during the adsorption-release process with weak hysteresis. This suggests that their pore channels were relatively unobstructed, and that the structure remained relatively stable during the cycling process (Fig. 4N).

In short, we successfully design and prepare a temporal-responsive hydrogels with rich pore structure, causing the continuous release of Sr^2+^ and the sustained release of Ber expected to match the energy metabolism cycle.

### 2.4 Biocompatibility of temporal hydrogels

**Fig. 5:**
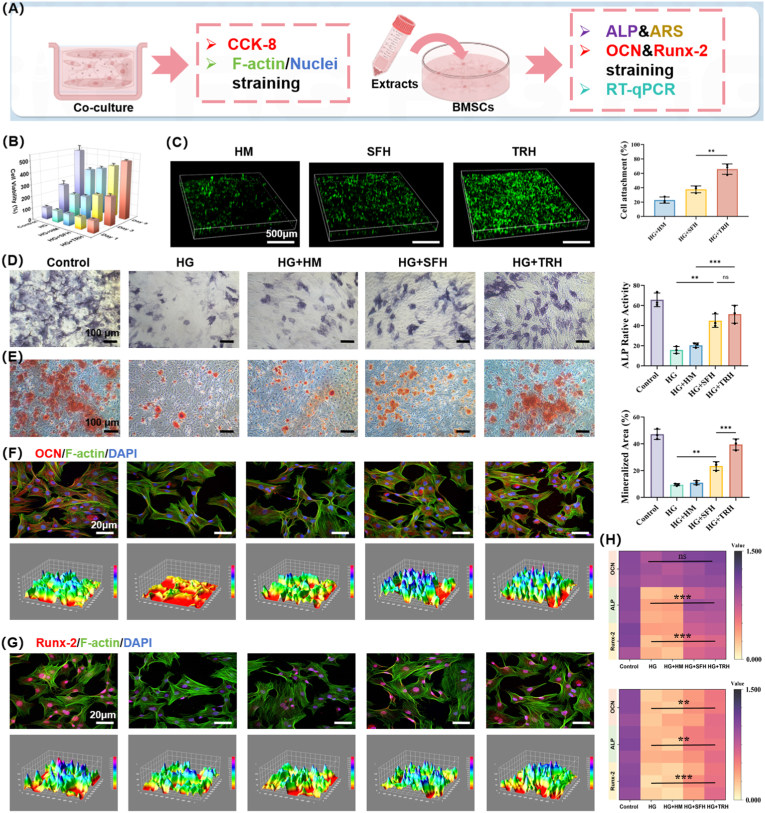
*In vitro* osteogenesis effect of different treatments. (A) Schematic illustration of the in vitro osteogenesis test. (B) CCK-8 assay for cellular viability in each group at 1, 3, and 5 d (n = 5). (C) Cytoskeletal F-actin (green) distribution of BMSCs cultured under different treatments for 24 h. The cell nuclei were counterstained with DAPI (blue). Bar = 500 μm. (D) ALP staining at day 7. Bar = 100 μm. (E) ARS staining at day 14. Bar = 100 μm. (F) IF images of BMSCs stained by OCN (red), phalloidin (green), and DAPI (blue) under different treatments for 7 d. Bar = 20 μm. (G) IF images of BMSCs stained by RUNX2 (red), phalloidin (green), and DAPI (blue) under different treatments for 7 d. Bar = 20 μm. (H) Relative mRNA expression of osteogenic-related genes (Runx2, ALP, OCN) at different time points (upper panel: day 7; lower panel: day 14; n = 3). Statistical analysis was performed for each gene at each time point using one-way ANOVA; significance levels are indicated in the corresponding quantitative panels.

BMSCs were cultured under high glucose (HG, 25 mM) as a diabetic-like cellular stress condition and treated with extracts of different hydrogels. The schematic workflow of the in vitro assay is shown in Fig. 5A. The CCK-8 results (Fig. 5B) demonstrated that the HG group exhibited significantly lower cell proliferation than the control group at all time points (p < 0.01), confirming that high-glucose stress inhibited BMSC proliferation. The HG + HM group showed no significant difference compared with the HG group, indicating that the hydrogel matrix itself had no apparent pro-proliferative or cytotoxic effect. The HG + SFH group showed higher OD values than the HG group on days 3 and 5 (p < 0.05), indicating that Sr-HA release improved cell proliferation. Importantly, the HG + TRH group exhibited the highest proliferation activity at all time points. On day 5, its OD value was significantly higher than those of the HG group (p < 0.001) and the HG + SFH group (p < 0.05), while showing no significant difference from the control group. These results demonstrate that the released functional components from TRH counteracted high-glucose-induced cellular dysfunction and restored BMSC proliferation capacity.

F-actin/DAPI staining visually confirmed the CCK-8 results. After 3 d of culture, both the control and HG + TRH groups showed densely packed and well-spread cells (Fig. 5C). In contrast, the HG and HG + HM groups showed markedly reduced cell density and shrunken morphology. The HG + SFH group showed better cell status than the HG group but remained inferior to the TRH group. These observations demonstrate the excellent biocompatibility of TRH and its ability to preserve cell viability under high-glucose conditions. In parallel, direct cell–hydrogel contact imaging was used to evaluate the cell-material interface, confirming that cells could attach and spread on the TRH surface. Therefore, direct-contact imaging and extract-based functional assays were used as complementary approaches to assess interface compatibility and release-mediated bioactivity, respectively [39].

### 2.5 Osteogenic capability of temporal hydrogels

We first evaluated alkaline phosphatase (ALP) activity, a key marker of early osteogenic differentiation (Fig. 5D). After 7 d of osteogenic induction, ALP activity in the HG group was significantly lower than that in the control group (p < 0.001). The HG + HM group showed only a slight, non-significant improvement. By contrast, ALP activity in the HG + SFH group was significantly higher than that in the HG group (p < 0.01). The HG + TRH group showed a further slight increase relative to the SFH group, although the difference was not statistically significant (p > 0.05). After 14 d of osteogenic induction, ARS staining revealed significant differences in late-stage mineralization among groups. The HG group showed only minimal calcium nodule formation (Fig. 5E), whereas the HG + SFH group showed a significantly greater number and area of mineralized nodules than the HG group (p < 0.01). The HG + TRH group exhibited the strongest mineralization capacity, with calcium nodule deposition markedly higher than that in all other groups (p < 0.001). To further explore the cellular and molecular basis of this temporal difference, we conducted additional analyses at both the protein and gene levels [40].

Immunofluorescence staining at day 7 revealed that RUNX2 nuclear intensity was elevated in both the SFH and TRH groups compared with the HG group, consistent with the ALP results (Fig. 5G). Notably, OCN staining already showed a stronger trend in the TRH group than in the SFH group at day 7, suggesting earlier entry into mid-to late-stage differentiation programs in TRH-treated cells (Fig. 5F). After 7 d of osteogenic induction, RUNX2 and ALP mRNA levels were significantly upregulated in both the HG + SFH and HG + TRH groups, with no significant difference between these two groups. After 14 d, OCN expression was significantly higher in the HG + SFH group than in the HG group; additionally, OCN expression in the HG + TRH group was markedly higher than that in the HG + SFH group (Fig. 5H).

Overall, TRH effectively alleviated the inhibitory effect of high-glucose stress on early osteogenic differentiation, partly through Sr^2+^-mediated osteogenic priming. Moreover, sustained Ber release may support mitochondrial metabolic recovery during later osteogenic maturation. Because ALP activity and ARS-detected mineralization are stage-dependent readouts affected by Sr^2+^/Ca^2+^ release, Ber release, cell proliferation, induction duration, and the hydrogel microenvironment, these osteogenic outcomes should be interpreted as the overall effect of the temporally coordinated TRH system rather than as a Ber-only response [41].

### 2.6 Angiogenic capacity of temporal hydrogels

**Fig. 6:**
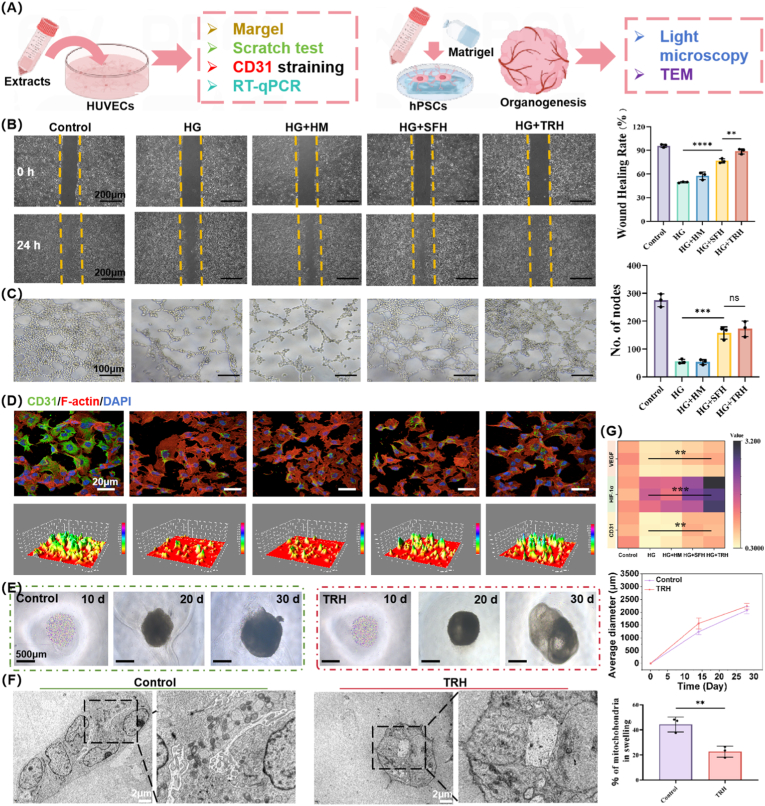
In vitro angiogenesis effect of different treatments. (A) Schematic illustration of the in vitro angiogenesis test. (B) Scratch tests of HUVECs under different treatments for 12 h. Bar = 200 μm. (C) Matrigel tube-forming assays of HUVECs under different treatments for 6 h. Bar = 100 μm. (D) IF images of HUVECs stained by CD31 (green), phalloidin (red), and DAPI (blue) under different treatments for 48 h. Bar = 20 μm. (E) Morphology of vascular organoids on days 10, 20, and 30. Bar = 500 μm. (F) TEM images of organoids. Bar = 2 μm. (G) Relative mRNA expression of angiogenesis-related genes (Hif-1α, CD31, VEGF; n = 3). Quantitative data are shown as mean ± SD, and statistical significance is indicated in the corresponding panels.

Endothelial dysfunction is a key early barrier to diabetic healing. The in vitro angiogenesis workflow is shown in Fig. 6A. In the scratch assay (Fig. 6B), the wound gap in the control group was nearly closed after 12 h. The healing rate of the HG group was significantly lower than that of the control group (p < 0.001), indicating that high glucose markedly inhibited HUVEC migration. No significant difference was observed between the HG + HM and HG groups. In contrast, the scratch-healing rates in the HG + TRH and HG + SFH groups were significantly higher than that in the HG group (p < 0.01), demonstrating that SFH and TRH alleviated the inhibitory effect of high glucose on endothelial migration.

After 6 h of culture on Matrigel, HUVECs in the control group formed intact, dense tubular networks (Fig. 6C). In contrast, the HG group exhibited sparse and fragmented tubular structures, with significant reductions in total tube length, branch number, and mesh number (p < 0.001). The HG + HM group showed no significant improvement. The HG + SFH group showed substantial recovery of tube formation, with all parameters significantly higher than those in the HG group (p < 0.01). Notably, the HG + TRH group performed better than the other treatment groups, forming tubular networks that more closely resembled those of the control group, although no significant difference was observed compared with the SFH group (p > 0.05).

Immunofluorescence staining (Fig. 6D) showed that, compared with the control group, the HG group had markedly reduced CD31 expression (green fluorescence) on the cell surface, together with a disorganized cytoskeletal structure (F-actin, red fluorescence). In the HG + SFH and HG + TRH groups, CD31 fluorescence signals were the strongest and most continuous among all groups, with predominant localization at cell-cell junctions and a characteristic peripheral staining pattern. To validate the pro-angiogenic capacity of the temporal hydrogels at the molecular level, we examined the expression of key angiogenic genes. CD31 immunofluorescence showed strong and continuous junctional localization in SFH- and TRH-treated cells, with significantly higher fluorescence intensity than in the HG or HM groups (p < 0.001; Fig. 6G). At the transcriptional level, HG downregulated CD31 and VEGF while upregulating HIF-1α, reflecting a hypoxic stress response. SFH and TRH restored CD31 expression (0.76 ± 0.04 and 0.87 ± 0.06 vs. 0.57 ± 0.05 in HG; p < 0.001) and increased HIF-1α and VEGF expression, supporting enhanced angiogenic signaling (Fig. 6G).

To further validate vascular function in a 3D context, we evaluated vascular organoids (Fig. 6E and F). Organoids cultured with TRH-conditioned medium displayed improved spheroid growth and lumen formation from days 10 to 30, and TEM confirmed tight junction structures and abundant mitochondria around the lumen. The control group exhibited lower vascular maturity, whereas the TRH group displayed more mature vascular-like structures. The average diameter was higher in the TRH group than in the control group (2238 ± 108 μm vs. 2079 ± 144 μm). Furthermore, the swelling rate was lower in the TRH group than in the control group (22.7% vs. 44.3%, p < 0.01). Collectively, these results indicate that TRH more effectively supports angiogenesis.

### 2.7 Osteogenic mechanism of temporal hydrogels

**Fig. 7:**
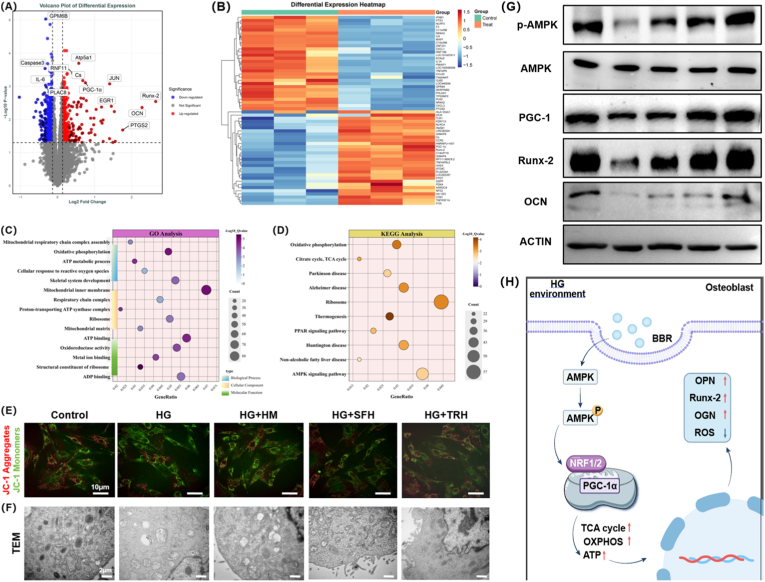
Transcriptome sequencing data validation. (A) Differential RNA expression profile between HG and TRH conditions, illustrated by volcano plot analysis. (B) Heatmap clustering of transcriptional alterations comparing HG and TRH samples. (C) GO functional annotation enrichment. (D) Enriched pathways identified via KEGG analysis. (E) JC-1-based fluorescence assessment of mitochondrial membrane potential. Scale bar = 10 μm. (n = 3). (F) Mitochondrial ultrastructure visualized by TEM. Scale bar = 2 μm.(n = 3). (G) Protein expression of PGC-1α-related pathways via Western blot.(n = 3). (H) Schematic of the proposed mechanism underlying TRH-mediated cellular energy regulation.

To dissect the molecular basis underlying TRH-enhanced late-stage osteogenesis under diabetic stress, we performed mRNA sequencing of BMSCs after 14 d of osteogenic induction in high glucose. Compared with the HG group, the TRH group showed 1285 differentially expressed genes (DEGs), including 687 upregulated and 598 downregulated genes (Fig. 7A). The volcano plot illustrates the distribution of these DEGs, with several key osteogenic and metabolism-related genes highlighted. Fig. 7B shows upregulated genes such as PGC-1α, Cs, Atp5a1, OCN, and Runx2, and downregulated genes such as Il6 and Casp3, which are associated with inflammation or apoptosis.

Gene Ontology (GO) enrichment analysis further revealed that TRH predominantly activated an energy-osteogenesis-coupled program (Fig. 7C). In the biological process (BP) category, enriched terms were mainly related to mitochondrial respiratory chain complex assembly, oxidative phosphorylation, ATP metabolic processes, cellular responses to reactive oxygen species, and skeletal system development. In the cellular component (CC) category, significant enrichments were observed in the mitochondrial inner membrane, respiratory chain complex, proton-transporting ATP synthase complex, and ribosome. In the molecular function (MF) category, major enrichments included ATP binding, oxidoreductase activity, metal ion binding, and structural constituents of the ribosome. KEGG pathway analysis further confirmed these findings, with upregulated genes significantly enriched in oxidative phosphorylation, citrate cycle (TCA cycle), Parkinson disease, Alzheimer disease, ribosome, thermogenesis, and PPAR signaling pathways (Fig. 7D).

Under diabetic conditions, a high-glucose environment impairs mitochondrial function, forcing MSCs to remain in a glycolysis-dependent proliferative state and hindering their transition to the high-energy-consuming osteogenic differentiation phase [42]. Genes encoding mitochondrial electron transport chain (ETC) complexes, including Ndufv2, Sdha, Cox7a2, and Atp5a1, were broadly upregulated in the TRH group. These data suggest that TRH improves mitochondrial oxidative phosphorylation capacity and provides more ATP to meet the energy demand of osteogenic differentiation and matrix mineralization. Importantly, PGC-1α, a master regulator of mitochondrial biogenesis and oxidative metabolism, was markedly elevated at the transcript level. Based on these data and the added p-AMPK/AMPK validation, we interpret the effect of TRH as being associated with activation of an AMPK/PGC-1α-related mitochondrial pathway rather than as definitive AMPK-dependent causality. Improved energy availability may then support osteogenic transcription and maturation programs, as indicated by upregulation of Runx2 and OCN.

To functionally validate whether TRH enhances mitochondrial energy metabolism, we performed a series of mitochondrial analyses. As shown by JC-1 staining (Fig. 7E), after 14 d of osteogenic induction, the red/green fluorescence ratio in the HG group was significantly lower than that in the control group (p < 0.01), indicating that hyperglycemia decreased mitochondrial membrane potential and impaired mitochondrial function. The mitochondrial membrane potential in the HG + TRH group was significantly restored compared with the other intervention groups, with a ratio of 0.89 ± 0.07, which was not significantly different from that of the control group (p > 0.05). This finding suggests that TRH protects against high-glucose-induced mitochondrial damage and improves mitochondrial function.

Transmission electron microscopy (TEM) provided ultrastructural evidence consistent with this functional recovery (Fig. 7F). In the control group, mitochondria exhibited intact morphology with clear and densely arranged cristae. In the HG group, mitochondria appeared markedly swollen, with disrupted or absent cristae and vacuolation. In contrast, mitochondria in the HG + TRH group showed improved morphology, with intact and orderly arranged cristae and a trend toward increased mitochondrial number. In the TRH group, the mitochondrial count recovered to 82.5 ± 8.3% of the control level (18 ± 5.3 vs. 13 ± 4.2 in the HG group, p < 0.05) (Fig. S1); meanwhile, mitochondrial cristae length recovered to 73.5 ± 4.8% of the control level (0.58 ± 0.02 μm/μm2 vs. 0.25 ± 0.03 μm/μm2 in the HG group, p < 0.05) (Fig. S2).

Western blotting further supported activation of the AMPK/PGC-1α-related mitochondrial osteogenesis pathway at the protein level (Fig. 7G and Fig. S3). Compared with HG, TRH increased the p-AMPK/AMPK ratio and PGC-1α protein expression, consistent with the RNA-seq results and supporting enhanced mitochondrial biogenesis/metabolic remodeling. TRH also elevated the osteogenic master regulator RUNX2 and the late-stage maturation marker OCN, indicating that mitochondrial metabolic recovery was accompanied by strengthened osteogenic differentiation and mineralization.

In addition, intracellular ATP and lactate assays were added to functionally evaluate cellular energy output and glycolysis-associated metabolic stress under high-glucose conditions. TRH restored ATP production and reduced lactate accumulation compared with the HG group, further supporting the conclusion that TRH improves mitochondrial energy competence while alleviating glycolysis-associated stress (Fig. S4).

Overall, transcriptomic, mitochondrial, and protein-level analyses support the central concept that TRH improves cellular energy metabolism under diabetic-like stress, particularly by enhancing mitochondrial oxidative phosphorylation-related programs. Sr^2+^ release promotes vascularization and creates a pro-regenerative niche, whereas Ber release is associated with activation of the AMPK/PGC-1α-related mitochondrial pathway and improved mitochondrial ultrastructure. These effects collectively increase ATP availability and support osteogenic differentiation and mineralization.

### 2.8 *In vivo* bone regeneration potential of temporal hydrogels

**Fig. 8:**
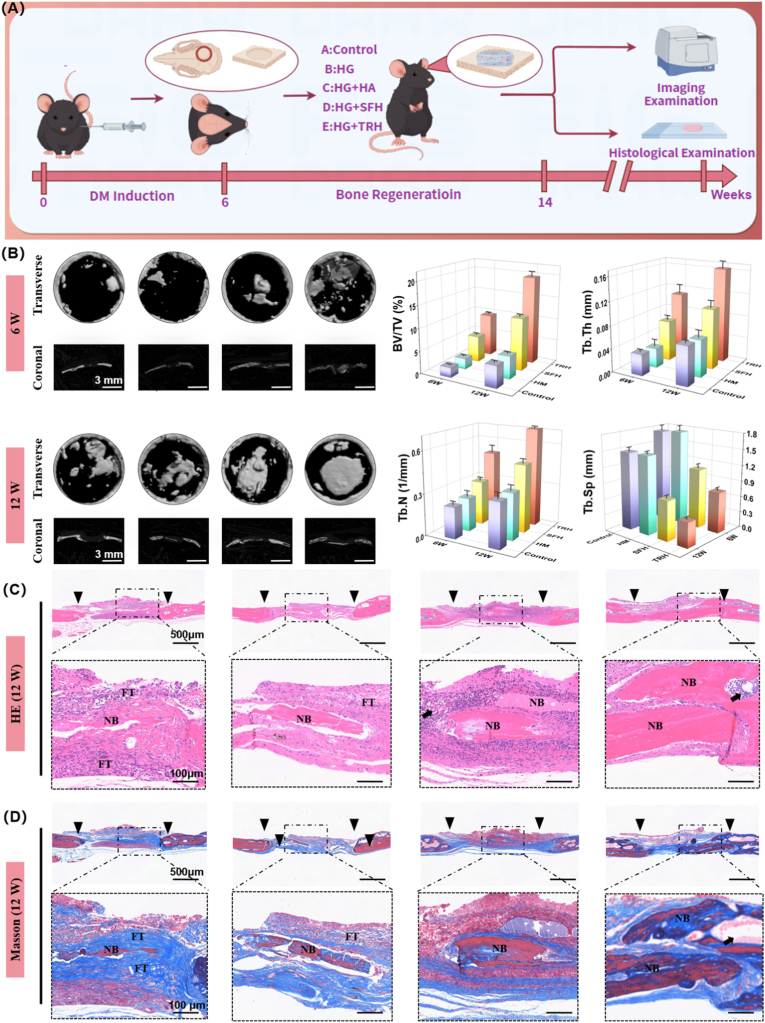
*In vivo* assessment of bone regeneration after scaffold implantation using Micro-CT and histological analyses. (A) Schematic overview of the experimental timeline and procedures. (B) Representative three-dimensional Micro-CT reconstructions of calvarial defects at 6- and 12-weeks post-implantation. Quantitative microarchitectural parameters of the newly formed bone, including bone volume fraction (BV/TV), trabecular number (Tb.N), thickness (Tb.Th), and separation (Tb.Sp). Histological evaluation by (C) H&E and (D) Masson's trichrome staining of the defect area.

To systematically evaluate the *in vivo* osteogenic performance of TRH, we first performed precise three-dimensional reconstruction and quantitative analysis of the bone defect region using micro-CT (Fig. 8A). Bone histomorphometry at postoperative week 6 showed that the HG + TRH group had the best performance across key parameters. Specifically, the bone volume fraction (BV/TV) in the HG + TRH group reached 9.47 ± 0.54% (Fig. 8B), which was significantly higher than that in the HG + SFH group (5.8 ± 0.45%; p < 0.05). In terms of microstructural parameters, the HG + TRH group showed significant advantages in trabecular number (Tb.N, 0.5 ± 0.053 1/mm) and trabecular thickness (Tb.Th, 0.11 ± 0.016 mm), whereas trabecular separation (Tb.Sp, 0.77 ± 0.067 mm) was significantly reduced. These results indicate that TRH had already begun to promote osteogenesis during the mid-term repair period.

Quantitative results at postoperative week 12 showed even more pronounced intergroup differences. BV/TV in the HG + TRH group increased further to 19.5 ± 1.15%, which was significantly higher than that in the other groups (p < 0.001). For bone microstructural parameters, the HG + TRH group achieved a Tb.N of 0.71 ± 0.016 1/mm and a Tb.Th of 0.16 ± 0.012 mm, while Tb.Sp decreased to 0.47 ± 0.049 mm (p < 0.001). These data clearly demonstrate the more pronounced osteogenesis-promoting efficacy of TRH during the late stage of repair. This is consistent with the temporal characteristics observed in the earlier mitochondrial-function validation experiments.

Histological analyses at week 12 further corroborated the micro-CT findings. H&E staining revealed that the HG + TRH group achieved robust defect bridging with structurally organized nascent trabecular bone and well-reconstituted marrow-like cavities, whereas the HG + SFH group still displayed residual fibrous tissue in the central defect (Fig. 8C). Masson's trichrome staining showed significantly increased new bone area in the HG + TRH group (68.3 ± 4.6%) compared with the HG + SFH group (37 ± 6.4%, p < 0.01), indicating more mature extracellular matrix formation (Fig. 8D and Fig. S5). Collectively, these results suggest that TRH not only increased bone matrix production but also promoted high-quality tissue maturation during late-stage repair.

### 2.9 Spatial colocalization analysis of characteristic markers

**Fig. 9:**
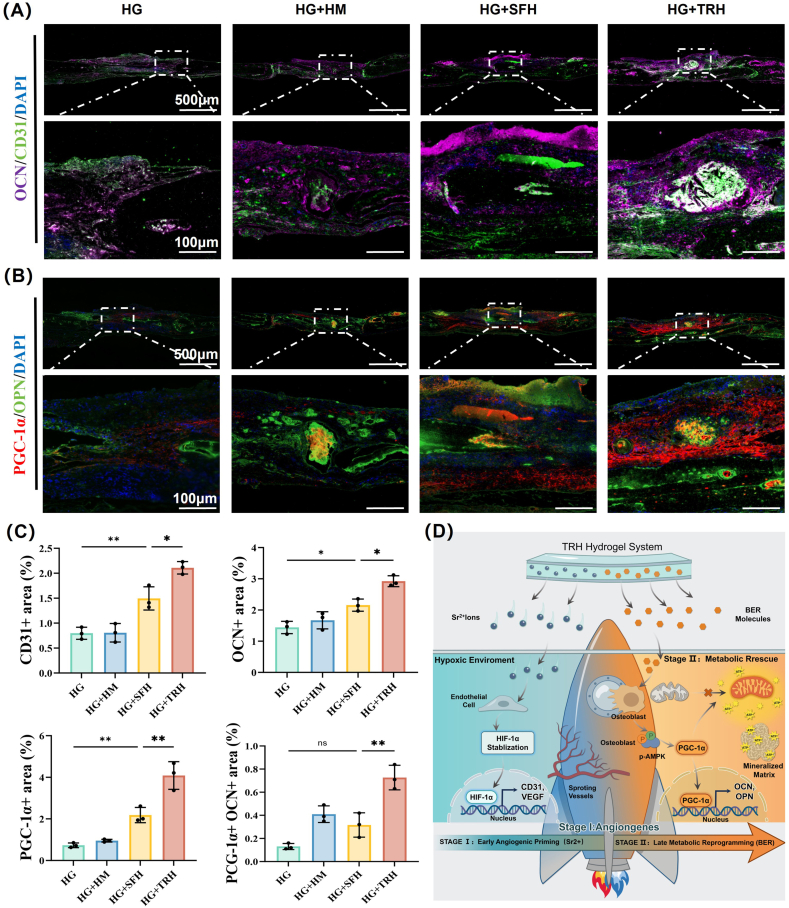
*In vivo* assessment of bone defect repair after scaffold implantation via immunofluorescence analysis. (A) Dual immunofluorescence staining for CD31 (green) and OCN (purple), with nuclei counterstained in blue. (B) Dual immunofluorescence staining for OPN (green) and PGC-1α (red), with nuclear staining in blue. Upper panel scale bar = 500 μm; lower panel scale bar = 100 μm. (C) Semiquantitative analysis of proteins related to osteogenesis and those related to the PGC-1α pathway. (D) Schematic diagram of temporal-responsive hydrogels regulating energy metabolism pathways.

To elucidate the mechanism at the cellular and molecular levels, we performed spatial colocalization analysis of key markers by double immunofluorescence staining (Fig. 9A and B). CD31/OCN double staining showed that the HG + TRH group had the highest CD31-positive area (2.13 ± 0.06%) and OCN-positive area (2.83 ± 0.07%) among all groups. These markers showed strong spatial colocalization, forming a typical vascular-osteogenic unit. This pattern was not evident in the other groups, indicating that TRH promoted vascular-bone cascade regeneration even under diabetic conditions.

Moreover, PGC-1α/OPN dual immunofluorescence staining (Fig. 9C) showed that the proportion of PGC-1α fluorescence intensity within OPN-positive osteoblasts reached 0.73 ± 0.05 in the HG + TRH group, which was significantly higher than that in the HG + SFH group (0.32 ± 0.06, p < 0.001). These data link mitochondrial functional status to cellular mineralization activity and provide *in vivo* evidence supporting the temporal energy hypothesis. The results suggest that, during late-stage repair, osteoblasts in the TRH group may achieve efficient mineralization partly through improved mitochondrial ATP synthesis capacity. This finding is consistent with the metabolic remodeling and ultrastructural improvements observed in vitro.

Based on these findings, we propose a temporal-responsive mechanistic model for diabetic bone regeneration. Continuous Sr^2+^ release enhances CD31-positive angiogenesis and improves the pro-regenerative niche, thereby indirectly supporting early osteogenic priming and differentiation. Sustained Ber release is associated with activation of the AMPK/PGC-1α-related mitochondrial pathway, strengthened mitochondrial oxidative metabolism and ATP availability, and increased osteogenic maturation/mineralization markers (OCN and OPN), thereby supporting late-stage differentiation and mineral deposition (Fig. 9D).

### 2.10 Discussion: scientific hypothesis and experimental validation

In this study, we developed and validated an injectable hydrogel based on a temporal energy-regulation strategy, providing an innovative materials-based solution to overcome impaired diabetic bone repair. The central concept of this study is to emulate and guide the natural temporal dynamics of energy metabolism during bone regeneration. Specifically, promoting glycolysis-dependent vascularization during the early proliferative phase and enhancing mitochondrial oxidative phosphorylation during the later differentiation/mineralization phase can mitigate the energy deficit underlying diabetic bone repair.

First, transcriptomic analysis of diabetic bone defects supported the design rationale that energy metabolism changes dynamically during repair. Second, in vitro experiments confirmed the temporal functionality of the material design. Early Sr^2+^ release promoted endothelial migration and tube formation by enhancing angiogenesis-related signaling, thereby establishing a vascularization foundation for regeneration. Meanwhile, sustained Ber release was associated with activation of the AMPK/PGC-1α-related mitochondrial pathway, improved ATP production, and enhanced osteogenic maturation. Finally, *in vivo* experiments in diabetic animal models validated the effectiveness of this strategy, demonstrating improved bone volume and microarchitecture.

Several limitations should be noted. First, an isotonic mannitol control was not included in the in vitro high-glucose experiments; therefore, although 25 mM glucose is widely used to establish diabetic-like cellular stress, the possible contribution of hyperosmolarity cannot be completely excluded. Second, the current ALP and ARS assays were performed at representative endpoints rather than as a full time-course analysis, and these stage-dependent readouts may be influenced by Sr^2+^/Ca^2+^ release, Ber release, cell proliferation, induction duration, and the hydrogel microenvironment. Therefore, the enhanced osteogenic activity should be interpreted as the overall effect of TRH rather than a direct quantitative correlation between Ber release kinetics and peak ALP or mineralization activity. Third, a complete free-Ber/Sr^2+^ single-agent matrix and AMPK inhibition/knockdown experiments were not performed; thus, the proposed AMPK/PGC-1α-related mechanism should be considered associative rather than definitive AMPK-dependent causality. Fourth, the actual *in vivo* release amounts of Ber and Sr-containing ions from the defect site were not directly quantified because local retrieval and separation of released components from the regenerating calvarial tissue are technically difficult. Therefore, the *in vivo* exposure was interpreted based on the nominal implantation dose and in vitro release profiles. Future studies will further clarify these quantitative and mechanistic relationships.

In summary, the principal innovation of this work lies in the proposed temporal energy strategy, which dynamically aligns with the differential energy demands of distinct cellular activities throughout the repair process. Based on this strategy, the TRH hydrogel successfully overcame healing stagnation caused by energy deficiency in the diabetic microenvironment through the coordinated action of early-stage pro-vascularization support and later-stage mitochondrial regulation. This provides a foundation for developing next-generation intelligent biomaterials for metabolic bone diseases.

## 3 Conclusion

In summary, we proposed and validated a temporal energy strategy to overcome impaired diabetic bone repair. Transcriptomic reanalysis revealed stage-dependent metabolic remodeling during diabetic healing. TRH provided Sr^2+^ release for up to 30 d and achieved delayed Ber delivery. At 30 h, TRH achieved a cumulative Ber release of only 48.7%, which was lower than that of the control group (87.2%). TRH restored endothelial migration and tubular networks under hyperglycemic conditions and improved mitochondrial membrane potential, ultrastructural integrity, and ATP production, thereby supporting osteogenic differentiation and mineralization. In a critical-sized diabetic calvarial defect model, TRH markedly increased bone volume and improved trabecular microarchitecture. Compared with the control group (4.8%), the bone volume fraction in the TRH group increased to 19.5%. These findings highlight temporal metabolic programming as a promising design principle for regenerative biomaterials and suggest that TRH is a potential therapeutic platform for diabetic bone defects.

## 4 Materials and methods

### 4.1 Materials

Chitosan (Deacetylation degree ≥95%), Carboxymethyl chitosan (CMCS, Degree of substitution: ≥80%), SrCl_2_, NaCl, NaHCO_3_, KCl, K_2_HPO_4_∙3H_2_O, MgCl_2_∙6H_2_O, CaCl_2_, and Na_2_SO_4_ were obtained from Aladdin, Shanghai, China. Bacterial cellulose (BC) was acquired from Guilin Qihong Technology Co., Ltd., China. Genipin was acquired from Linchuan Zhixin Bio-Technology Co., Ltd., China. Berberine hydrochloride (Ber) and sodium periodate (NaIO_4_, purity ≥99.5%) were purchased from Shanghai Macklin Biochemical Technology Co., Ltd, China. Acetic acid and glutaraldehyde were provided by Chengdu Kelong Chemical Reagent Factory, China.

### 4.2 Sample preparation

100 mg of SrCl_2_ was added to the 4×SBF solution (Table S1.). The mixture was then got at 37 °C for 48 h until a white precipitate formed at the bottom of the container. Subsequently, the white mineralized particles were collected by centrifugation and washed twice to remove excess salts. The product was then freeze-dried for 3 d to obtain strontium doped hydroxyapatite (Sr-HA).

A 2 wt% chitosan solution was prepared, and 100 mg of berberine hydrochloride was added per 100 mL of the solution. 0.6 mL of Span-80 was added in 30 mL of liquid paraffin. The mixture was then stirred using a magnetic stirrer at 50 °C and 1200 rpm for 10 min until a homogeneous state was achieved. The pre-prepared drug-loaded chitosan solution was added to the aforementioned system at a dripping rate of 2 drops per second (2 d/s) under continuous stirring to form a stable emulsion. Subsequently, 0.5 mL of glutaraldehyde solution (25%) was added to the emulsion system, and stirring was continued for 1.5 h to achieve thorough cross-linking of the drug-loaded chitosan. Ethanol was added to demulsify the system, and the mixture was continuously maintained in an ice-water bath for 30 min. After the reaction was complete, centrifugation was carried out to obtain berberine-loaded chitosan microspheres.

3 g of carboxymethyl chitosan was added to 97 mL of deionized water and dissolved to obtain a 3 wt% carboxymethyl chitosan solution. Bacterial cellulose was selectively oxidized with sodium periodate solution for 12 h to obtain oxidized bacterial cellulose (OBC). 7 mL of carboxymethyl chitosan solution was mixed with 3 mL of OBC for initial dynamic cross-linking, resulting in the formation of a hydrogel precursor. Then 10 mg of drug-loaded microspheres, 10 mg of Sr-HA, and 200 μL of Genipin solution (20 mg/mL) were added for secondary cross-linking. The resulting sample was named as a time-responsive hydrogel (TRH). For comparison, a sample prepared following the same procedure but without drug-loaded microspheres and Sr-HA was designated as the hydrogel matrix (HM). The sample loaded with Sr-HA was designated as a single-function hydrogel (SFH).

### 4.3 Sample characterization

Field-emission scanning electron microscopy (FE-SEM; ZEISS Sigma 360, Germany) was used to examine the morphology of the hydrogels. Elemental mapping for C, N, O, Ca, P, Sr, and Cl in the hydrogels was carried out using X-ray energy-dispersive spectroscopy (EDS; OXFORD Xplore, UK). To determine the water absorption rate, freeze-dried hydrogels of the same quality were immersed in PBS (pH 7.4) until they reached swelling equilibrium. The water absorption rate was calculated as the ratio of the weight gain after complete swelling to the initial dry weight. The swelling ratio was calculated as (W_t_-W_0_)/W_0_×100%, where W_0_ and W_t_, are the dry weight and swollen weight at time t, respectively. The swelling process of temporal-responsive hydrogels was fitted using the Korsmeyer–Peppas model and the pseudo-second-order kinetic model. The Korsmeyer–Peppas model was calculated according to equations (1) and (2).

(1) $$F=\frac{S}{S_{\infty }}=k_{1}t^{n}$$

(2) $$\mathrm{InF}=In\left(\frac{S_{t}}{S_{\infty }}\right)={Ink}_{1}+nInt$$

Where S is the water absorption ratio (swelling ratio) at time t (g/g); $S_{\infty }$ is the equilibrium swelling ratio (ESR) (g/g); k_1_ is the swelling rate constant; and n is the diffusion exponent

Quasi-second-order kinetic model was calculated according to equations (3) and (4).

(3) $$\frac{dS}{dt}={k_{2}\left(S_{\infty }-S\right)}^{2}$$

(4) $$\frac{t}{S}=\frac{1}{\left(k_{2}S_{\infty }^{2}\right)}+\frac{1}{S_{\infty }}t$$

Where K_2_ is the Quasi-second-order swelling rate constant.

The weight remaining rate of hydrogels in PBS in the first 30 d was counted. A rotational rheometer (Physica MCR-92, Anton Paar, Austria) was used to investigate the storage modulus (G′) and loss modulus (G″) of the hydrogels. The compressive properties of the hydrogel were tested using a universal testing machine (5967, Instron, USA), with samples measuring 26 mm in diameter and 10 mm in height. The crystal structure of the hydrogels was examined via X-ray diffraction (XRD; Rigaku SmartLab SE, Japan) over a 2θ range of 10° to 80°. Transmission electron microscopy (TEM; JEOL JEM-F200, Japan) was employed to investigate the lattice arrangement of the hydrogels. Fourier transform infrared (FT-IR) spectra were acquired on a Thermo Nicolet IS5 (USA) spectrometer over the range of 550 to 1800 cm^−1^. The drug release profile was monitored with a UV–visible spectrophotometer (UV–vis; Lambda 750 s, USA). The detection wavelength is 347 nm. The release kinetics of Ca^2+^ and Sr^2+^ from TRH were assessed by inductively coupled plasma optical emission spectrometry (ICP-OES; Agilent 5100 SVDV, USA).

High-performance liquid chromatography (HPLC) was used to determine the release profile of berberine during the first 14 d. The analysis was performed using a Dionex UltiMate 3000 HPLC system equipped with a C18 column (250 mm × 4.6 mm, 5 μm). The column temperature was maintained at 25 °C. The mobile phase consisted of 0.03 mol/L potassium dihydrogen phosphate solution and acetonitrile (60:40, v/v), with a flow rate of 1.0 mL/min. Detection was carried out at 347 nm, and the injection volume was 20 μL. Prior to analysis, the sample solution was filtered through a 0.45 μm membrane and directly injected into the system. The pore structure of the hydrogel was analyzed by specific surface area and pore size (BET) measurements. The samples were pre-degassed at 70 °C for 8 h, and then subjected to isothermal adsorption–desorption testing at the liquid nitrogen saturation temperature of 77 K. The specific surface area was calculated using the BET method, and the pore size distribution was determined by non-local density functional theory (NLDFT).

### 4.4 Transcriptome data analysis

Transcriptomic data of diabetic bone defect tissues from the public database GSE76364 were analyzed. According to the GEO metadata, fracture callus samples were collected at post-fracture day 5 and day 11, which were defined in this study as the early and later repair stages, respectively. The expression matrix was obtained by downloading the data from the GEO database using the GEOquery package in R. Principal component analysis (PCA) was first performed to assess the overall transcriptomic variance. Differential gene expression analysis between the later-stage and early-stage groups was conducted using the limma package, with thresholds set at |log2 fold change (FC)| > 0.5 and adjusted P value < 0.05. Differentially expressed genes were visualized using volcano plots and hierarchical clustering heatmaps. KEGG pathway enrichment analysis was performed using the ClusterProfiler package, with statistical significance set at P < 0.05. Finally, GSEA was applied to evaluate coordinated pathway changes based on gene-expression ranking (later stage vs. early stage).

### 4.5 Biocompatibility assessment

Primary mouse BMSCs were isolated following the methodology described in our previous work [43]. Cells were cultured in high-glucose (HG, 25 mM) medium to establish a diabetic-like cellular stress condition. A CCK-8 assay was performed to evaluate the cytocompatibility of temporal hydrogels. MSCs were seeded in 96-well plates at a density of 5 × 10^3^ cells per well. After cell attachment, the medium was replaced with HG medium containing hydrogel extracts. After 1, 3, and 5 d of culture, 10 μL of CCK-8 reagent (Beyotime, China) was added to each well, followed by additional incubation for 2 h. Absorbance at 450 nm was measured using a microplate reader (BioTek, USA). Five parallel replicates were used for each group. The relative cell growth rate (RGR) was calculated as follows: RGR (%) = (OD experimental group/OD control group) × 100%.

Subsequently, a cytoskeleton staining kit (Beyotime, China) was used for visual assessment of cell viability. BMSCs were seeded in 24-well plates and co-cultured with hydrogel extracts for 3 d. The medium was removed, and after fixation and permeabilization, FITC-labeled cytoskeleton staining reagent was added and incubated for 20 min at 37 °C in the dark. DAPI staining was then performed, and the samples were washed. Images were acquired using a confocal laser scanning microscope (CLSM, Nikon A1, Japan). For direct cell-hydrogel contact evaluation, cells were seeded on the hydrogel surface and stained with F-actin/DAPI to observe cell attachment and spreading at the material interface.

### 4.6 *In vitro* osteogenic differentiation assessment

Sterilized hydrogel extract was mixed with osteogenic induction medium for subsequent experiments [44]. BMSCs were cultured under high-glucose (25 mM) conditions during osteogenic induction. BMSCs were seeded in 24-well plates, and after 7 d of osteogenic induction, the cells were fixed with 4% paraformaldehyde. Subsequently, BCIP/NBT substrate chromogen solution (Beyotime, China) was added for ALP staining. For BMSCs undergoing 14 d of osteogenic induction, cells were fixed with 4% paraformaldehyde and incubated with 2% Alizarin Red S solution for 20 min to detect calcium nodules. Images were acquired under a microscope.

BMSCs were seeded in confocal dishes and subjected to 7 d of osteogenic induction. After fixation and permeabilization, the cells were blocked with 5% BSA. BMSCs were then incubated with anti-RUNX2 and anti-OCN primary antibodies at 4 °C overnight, followed by incubation with corresponding Alexa Fluor 488- or 594-conjugated secondary antibodies and DAPI. Images were acquired using a CLSM, and mean fluorescence intensity was quantified with ImageJ software.

After 7 or 14 d of osteogenic induction, total RNA was extracted from BMSCs using TRIzol and reverse-transcribed into cDNA. Amplification was performed on a real-time quantitative PCR instrument using SYBR Green. The detected genes included the early osteogenic transcription factor RUNX2, ALP, and the late osteogenic differentiation and mineralization marker OCN. GAPDH was used as the internal reference, and relative mRNA expression levels were calculated using the 2^(-ΔΔCt) method (n = 3).

### 4.7 Vasculature assessment

Human umbilical vein endothelial cells (HUVECs) were purchased from the Cell Bank of the Chinese Academy of Sciences. HUVECs were cultured in DMEM-F12 medium and seeded into 12-well plates at an appropriate density. After the cells reached full monolayer confluence, scratches were created using a sterile 200 μL pipette tip. After washing with PBS, the medium was replaced with conditioned media from the respective experimental groups. Scratch images were captured at 0 and 12 h using an inverted microscope. The scratch-healing rate was calculated using ImageJ software as follows: healing rate (%) = [(A_0_ - A_12_)/A_0_] × 100%.

Matrigel matrix (200 μL) was added to each well of a pre-chilled 48-well plate (−20 °C) and allowed to polymerize at 37 °C for 30 min. HUVECs were resuspended in conditioned media from the respective experimental groups and seeded onto the polymerized Matrigel. After 6 h of culture, images were acquired using an inverted microscope. Tubular structures, including total tube length, junction number, and mesh number, were quantified using ImageJ with the Angiogenesis Analyzer plugin.

HUVECs were then seeded in confocal dishes and co-cultured with conditioned media for 48 h. After fixation with 4% paraformaldehyde, permeabilization with 0.1% Triton X-100, and blocking with 5% BSA, cells were incubated with an anti-CD31 primary antibody (1:200) at 4 °C overnight. This was followed by incubation with an Alexa Fluor 488-conjugated secondary antibody (1:500), phalloidin (for F-actin staining), and DAPI (for nuclear staining) at room temperature for 1 h in the dark. Images were acquired using a confocal laser scanning microscope, and the mean fluorescence intensity of CD31 was quantified with ImageJ software to evaluate CD31 expression.

After HUVECs were co-cultured with the respective conditioned media for 48 h, total RNA was extracted using TRIzol and reverse-transcribed into cDNA. Amplification was performed on a real-time quantitative PCR instrument using SYBR Green. The detected genes included CD31, HIF-1α, and VEGF-A. GAPDH was used as the internal reference, and relative RNA expression levels were calculated using the 2^(-ΔΔCt) method (n = 3).

### 4.8 Mechanism assessment of osteogenesis

BMSCs were subjected to osteogenic induction under high-glucose (25 mM) diabetic-like stress conditions for 14 d and divided into two groups: HG and HG + TRH (n = 3). Total RNA was extracted using TRIzol. RNA samples were subjected to mRNA sequencing using the Illumina NovaSeq 6000 platform, with gene expression levels quantified by FPKM values. Differentially expressed genes (DEGs) were screened as follows: volcano plots were used to display the genome-wide distribution of up- and downregulated genes; GO enrichment analysis was conducted across BP, CC, and MF categories; and KEGG pathway enrichment analysis was performed using the clusterProfiler R package.

BMSCs were osteogenically induced in conditioned medium for 14 d. Staining was performed using JC-1 dye (Beyotime, China). In normal cells, JC-1 forms J-aggregates in the mitochondrial matrix that emit red fluorescence; when mitochondrial membrane potential decreases, JC-1 exists as J-monomers that emit green fluorescence. The red/green fluorescence ratio was imaged by confocal laser scanning microscopy and quantitatively analyzed.

Intracellular ATP content and lactate release were measured using commercial assay kits according to the manufacturers’ instructions to evaluate cellular energy output and glycolysis-associated metabolic stress. For Western blotting, total proteins were extracted from BMSCs after different treatments, separated by SDS-PAGE, transferred to PVDF membranes, and incubated with antibodies against p-AMPK, AMPK, PGC-1α, RUNX2, OCN, and β-actin.

### 4.9 *In vivo* bone regeneration

All animal experimental procedures were approved by the Animal Ethics Committee and conducted in accordance with international guidelines for the care and use of laboratory animals (XJTUAE20-2102). Six-week-old male genetically diabetic BKS-DB mice were used for the diabetic bone defect experiments, while age-matched wild-type mice served as non-diabetic controls where indicated. Blood glucose and body weight were monitored before surgery and during postoperative follow-up to confirm sustained diabetic/obese phenotypes. Only BKS-DB mice with blood glucose levels higher than 16.7 mmol/L were included in the diabetic groups. A critical-sized calvarial bone defect model (3 mm in diameter) was created in all mice. Immediately after defect creation, 20 μL of the corresponding hydrogel precursor or vehicle was injected into each defect to fill the cavity in situ. The mice were randomly assigned to five groups (n = 8): Control, HG, HG + HM, HG + SFH, and HG + TRH. At 6 and 12 weeks after surgery, four mice from each group were randomly euthanized to collect calvarial samples, which were scanned using a high-resolution micro-CT scanner. The defect area was reconstructed three-dimensionally using the accompanying software, and the following parameters were quantitatively analyzed: bone volume fraction (BV/TV, %) [45], trabecular number (Tb.N, 1/mm), trabecular thickness (Tb.Th, mm), and trabecular separation (Tb.Sp, mm).

### 4.10 Immunohistochemical analysis

At 12 weeks after surgery, mouse calvarial specimens were harvested and fixed in 4% paraformaldehyde, decalcified with EDTA, embedded in paraffin, and sectioned at 4 μm thickness. Sequential staining was performed as follows: H&E staining, Masson's trichrome staining [38,46], Sirius red staining, and immunofluorescence staining, including CD31/OCN and PGC-1α/OPN co-staining. Quantitative analyses were then conducted using ImageJ software to evaluate CD31-positive vessel density (vessels/mm^2^), OCN-positive area (%), and mean PGC-1α fluorescence intensity.

### 4.11 Statistical analysis

All data are presented as means ± standard deviations. One-way analysis of variance was used for statistical analysis unless otherwise stated. Sample sizes are indicated in the corresponding figure legends. Statistical significance is denoted as follows: ∗p < 0.05, ∗∗p < 0.01, ∗∗∗p < 0.001, and ∗∗∗∗p < 0.0001.

## CRediT authorship contribution statement

**Kai Jiang:** Conceptualization, Data curation, Investigation, Software, Writing – original draft, Writing – review & editing. **Kai Wang:** Conceptualization, Data curation, Funding acquisition, Supervision, Writing – original draft, Writing – review & editing.

## Declaration of competing interest

The all authors declare no conflict of interest.

## Data Availability

Data will be made available on request.
