# Supplementary material for: Temporal-responsive hydrogels reprogramming energy metabolic pathway in the bone-angiogenic cascade for diabetic bone regeneration
Source: Mater Today Bio. 2026 Jun 24;39:103394. doi: 10.1016/j.mtbio.2026.103394 (PMC13324684; doi:10.1016/j.mtbio.2026.103394)
Supplement: Multimedia component 1 [file mmc1.docx]

**Supporting** **Information**

**Temporal-Responsive Hydrogels Reprogramming Energy Metabolic Pathway in the Bone-Angiogenic Cascade for Diabetic Bone Regeneration**

Kai Jiang (江凯) ^a^, Kai Wang (王凯) ^b,^ *

^a^ Department of Critical Care Medicine, Sichuan Provincial People's Hospital, University of Electronic Science and Technology of China, Chengdu, 610054, China.

^b^ Department of Spine Surgery, Honghui Hospital, Xi'an Jiaotong University, Xi'an, 710054, China.^^[[1]](#footnote-0)^^

**Table S1.** Ion concentrations in prepared 4×SBF mediums.

| Ions (mM) | Cl^−^ | Na^+^ | Ca^2+^ | HPO_4_^2-^ | K^+^ | HCO_3_^−^ | Mg^2+^ | SO_4_^2−^ |
| --- | --- | --- | --- | --- | --- | --- | --- | --- |
| 4×SBF | 591.2 | 568 | 10 | 4 | 20 | 16.8 | 6 | 2 |

**Table S2.** Korsmeyer-Peppas model kinetic equations and kinetic parameters of different samples.

| Sample Name | Swelling Kinetics Equation | R^2^ | n | Ink_1_ |
| --- | --- | --- | --- | --- |
| HM | ln F=0.1547 ln t – 0.5564 | 0.9591 | 0.1547 | – 0.5564 |
| SFH | ln F=0.0946 ln t – 0.3771 | 0.9860 | 0.0946 | – 0.3771 |
| TRH | ln F=0.1087 ln t – 0.4684 | 0.9849 | 0.1087 | – 0.4684 |

**Table S3. Quasi-second-order kinetic model equations and kinetic parameters of different samples.**

| Sample Name | Swelling Kinetics Equation | R^2^ | S_∞_ | k_2_ |
| --- | --- | --- | --- | --- |
| HM | t/S=0.0445 t + 0.0821 | 0.9921 | 22.4211 | 0.02423 |
| SFH | t/S=0.0420 t + 0.0413 | 0.9983 | 24.8750 | 0.03913 |
| TRH | t/S=0.0427 t + 0.0473 | 0.9982 | 25.5909 | 0.03228 |

**Table S4.** Primer sequences used for RT-qPCR：Forward (F) and reverse (R) primer sequences (5′–3′) for rat genes involved in angiogenesis (VEGF, HIF-1α, PDGF, CD31), mitochondrial biogenesis/metabolism (PGC-1α), and osteogenesis (OPN, OCN, Runx2, COL-1). GAPDH was used as the housekeeping gene.

| **ID** | **primer name** | **primer sequence (5'to3')** |
| --- | --- | --- |
| 1 | VEGF-F | ATCGAGTACATCTTCAAGCCAT |
| 1 | VEGF-R | GTGAGGTTTGATCCGCATAATC |
| 2 | HIF-1α-F | GACCTGTGTGAGATCGACCA |
| 2 | HIF-1α-R | GTTGGTTTGGACGCCACTTC |
| 3 | PDGF-F | CCGTAACACATTTAGAAGCCAG |
| 3 | PDGF-R | CATCAAGCTACAACTTCAAGCA |
| 4 | CD31-F | CGTTGTCATTGGAGTGGTCAT |
| 4 | CD31-R | GAGTCGTAATGGCTGTTGGTT |
| 5 | PGC-1α-F | GAAAAAGCTTGACTGGCGTC |
| 5 | PGC-1α-R | GCAGCACACTCTATGTCACTC |
| 6 | OPN-F | CCAGCCAAGGACCAACTACA |
| 6 | OPN-R | AGTGTTTGCTGTAATGCGCC |
| 7 | OCN-F | GGCGCTACCTGTATCAATGG |
| 7 | OCN-R | GTGGTCAGCCAACTCGTCA |
| 8 | Runx-2-F | CCGAGACCAACCGAGTCATTTA |
| 8 | Runx-2-R | AAGAGGCTGTTTGACGCCAT |
| 8 | COL-1-F | CCCAGCGGTGGTTATGACTT |
| 8 | COL-1-R | TCGATCCAGTACTCTCCGCT |
| 9 | GAPDH-F | CCGAGACCAACCGAGTCATTTA |
| 9 | GAPDH-R | GATGGTGATGGGTTTCCCGT |


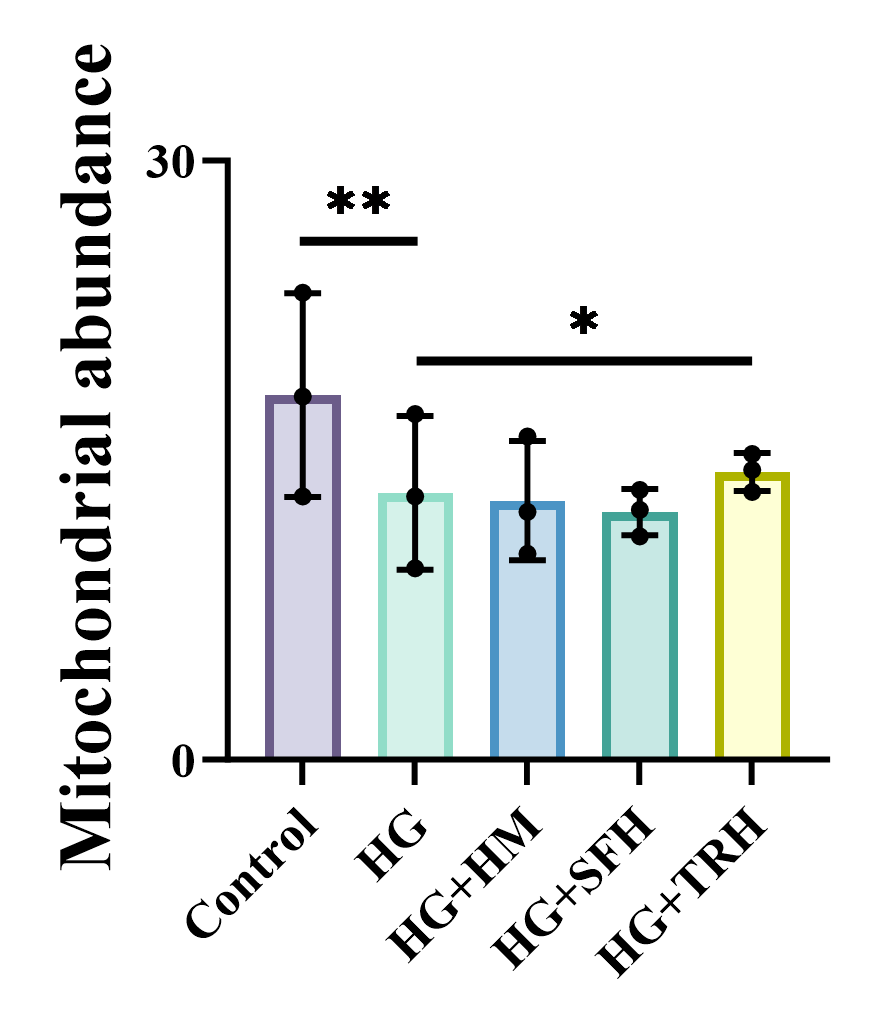


**Figure S1.** Quantification of mitochondrial abundance：Mitochondrial abundance (number of mitochondria per cell, quantified from TEM micrographs) in cells cultured under control conditions or high glucose (HG) with different treatments (HG, HG+HM, HG+SFH, HG+TRH). Data are shown as mean ± SD; dots represent individual samples (n = 3). Statistical significance is indicated by horizontal bars (*p < 0.05, **p < 0.01).


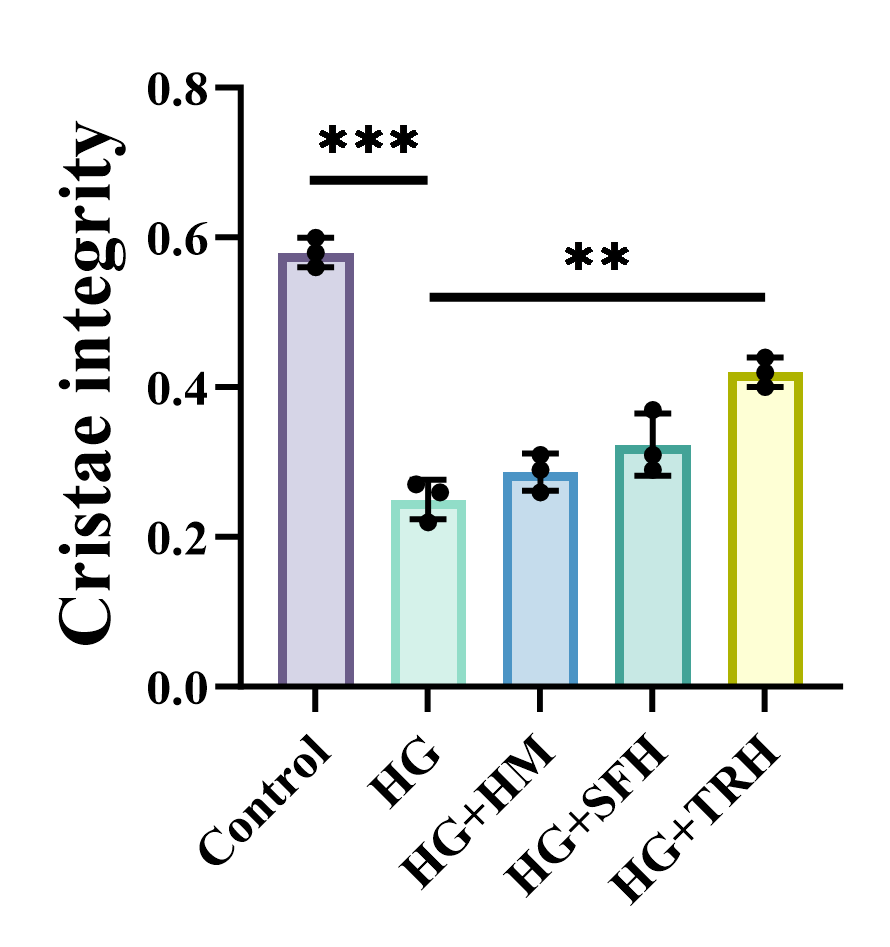


**Figure S2.** Quantification of mitochondrial cristae integrity：Cristae integrity quantified from TEM micrographs as cristae length density (μm/μm²) in cells cultured under control conditions or high glucose (HG) with different treatments (HG, HG+HM, HG+SFH, HG+TRH). Data are shown as mean ± SD; dots represent individual samples (n = 3). Statistical significance is indicated by horizontal bars (**p < 0.01, ***p < 0.001).


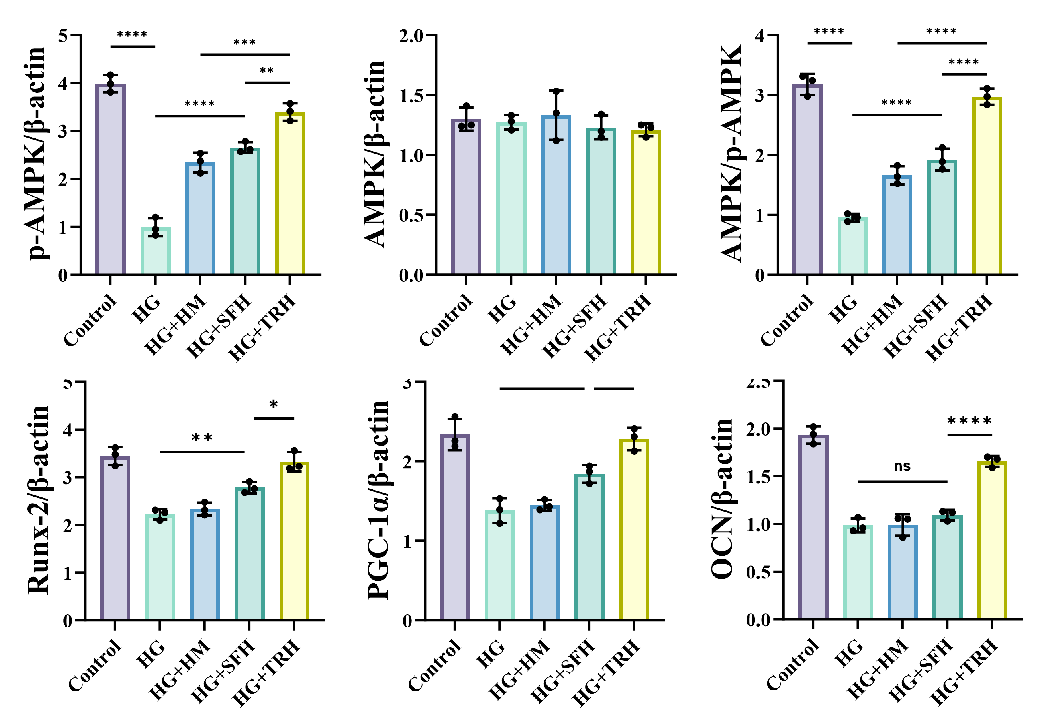


**Figure S3.** Representative pictures of Western blot analysis of BMSC cells (PCG-1α，Runx-2，OCN). Data are shown as mean ± SD; dots represent individual samples (n = 3). Statistical significance is indicated by horizontal bars (*p < 0.05, **p < 0.01).


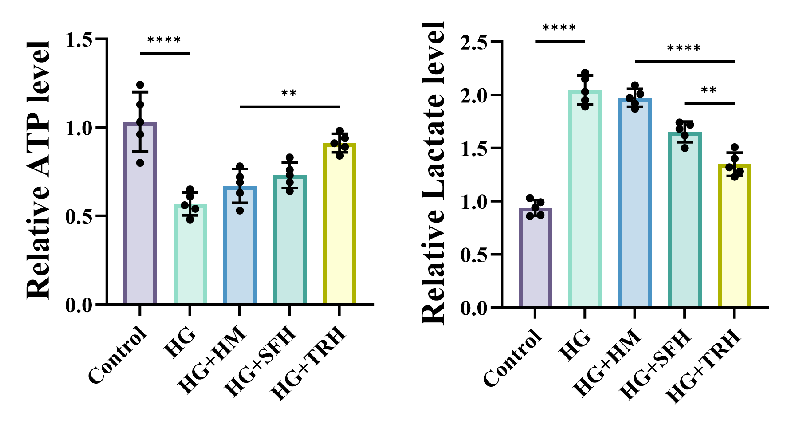


**Figure S4.** ATP content and Lactate release in cells (n = 5). Statistical significance is indicated by horizontal bars (*p < 0.05, **p < 0.01).


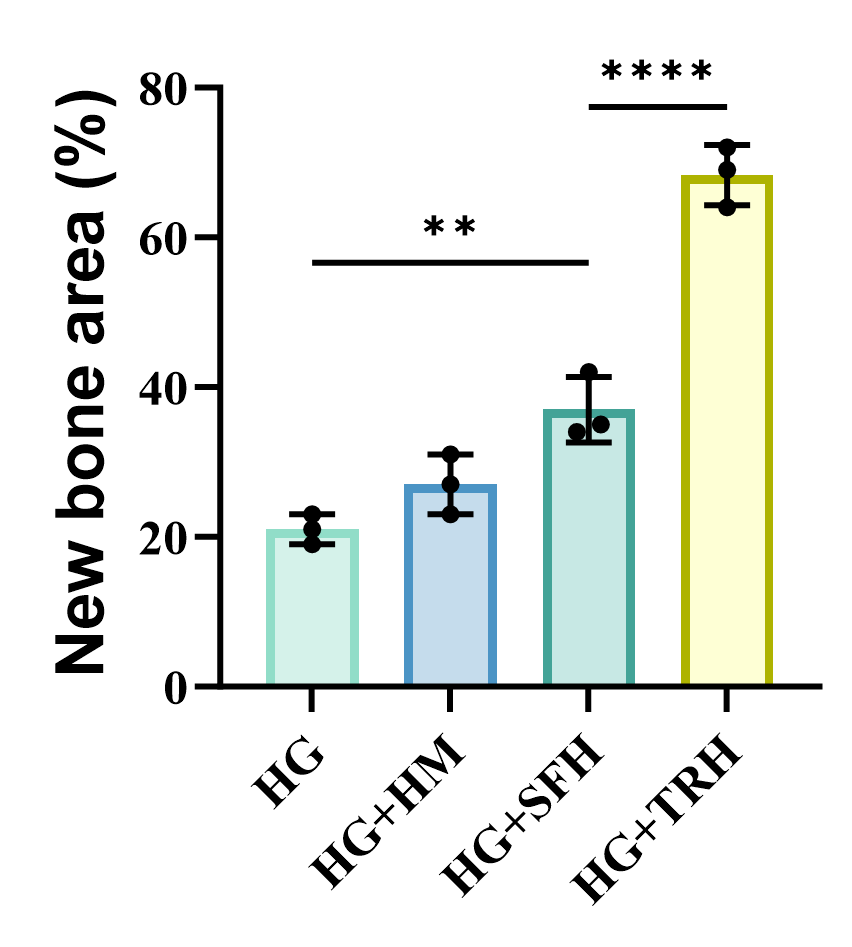


**Figure S5.** The ratio of regenerated bone area to bone defect in Masson staining (n = 3). Statistical significance is indicated by horizontal bars (*p < 0.05, **p < 0.01).

1. First author: Kai Jiang

   * Corresponding author: Kai Wang

   E-mail addresses: drwangkai0913@163.com (K. W.) [↑](#footnote-ref-0)
